# Evaluation of Genotoxicity of Water and Ethanol Extracts from *Rhus verniciflua* Stokes (RVS)

**DOI:** 10.5487/TR.2008.24.2.151

**Published:** 2008-06-01

**Authors:** Ji-Young Kim, Se-Wook Oh, Daeseok Han, Michael Lee

**Affiliations:** 18 Korea Institute of Toxicology, KRICT, Daejeon, 305-600 Korea; 28 Korea Food Research Institute, Sungnam, 463-746 Korea; 38 Department of Biology, College of Natural Sciences, University of Incheon, 177 Dowhadong, Nam-gu, Incheon, 402-749 Korea

**Keywords:** *Rhus verniciflua* Stokes (RVS), Ames assay, Chromosomal aberration test, Micronucleus assay, Genotoxicity

## Abstract

*Rhus verniciflua* Stokes (RVS), one of traditional medicinal plants in Asia, was found to have pharmacological activities such as antioxidative and antiapoptotic effects, raising the possibility for the development of a novel class of anti-cancer drugs. Thus, potential genotoxic effects of RVS in three short-term mutagenicity assays were investigated, which included the Ames assay, *in vitro* Chromosomal aberration test, and the *in vivo* Micronucleus assay. In Ames test, the addition of RVS water extracts at doses from 313 up to 5000 mg/plate induced an increase more than 2-fold over vehicle control in the number of revertant colonies in TA98 and TA1537 strains for detecting the frame-shift mutagens. The similar increase in reversion frequency was observed after the addition of RVS ethanol extracts. To assess clastogenic effect, *in vitro* chromosomal aberration test and *in vivo* micronucleus assay were performed using Chinese hamster lung cells and male ICR mice, respectively. Both water and ethanol extracts from RVS induced significant increases in the number of metaphases with structural aberrations mostly at concentrations showing the cell survival less than 60% as assessed by *in vitro* CA test. Also, there was a weak but statistically significant increase in number of micronucleated polychromatic erythrocytes (MNPCEs) in mice treated with water extract at 2000 mg/kg while ethanol extracts of RVS at doses of up to 2000 mg/kg did not induce any statistically significant changes in the incidence of MNPCEs. Therefore, our results lead to conclusion that RVS acts as a genotoxic material based on the available *in vitro* and *in vivo* results.

## INTRODUCTION

*Rhus verniciflua* is a species of family Anacardiaceae that grows in regions of Korea, China and Japan. The plant contains toxic substances that can cause severe irritation to some people. Nevertheless RVS has traditionally been used as a medicinal ingredient for the therapy of stomach and uterine cancer (Kim *et al.*, 2006) and for healing and treating hepatic and inflammatory diseases (Kim, 1996). Actually, several studies indicated that ethanol extracts of the plant have pharmacological activities such as antioxidative, and antiapoptotic effects (Lee *et al.*, 2002; Lim *et al.*, 2001), implying that *Rhus verniciflua* Stokes (RVS) might contain functional substances with multiple biological activities. Additionally, it was also suggested that the flavonoid fractions extracted from RVS could be applied to the material of functional food for enhancing the sexual function (Na *et al.*, 2005).

Despite its therapeutic potential, so far there have been no studies on the likely genotoxic effects of *Rhus verniciflua* Stokes (RVS). Genotoxicity assays can be defined as *in vitro* and *in vivo* designed to detect compounds, which include genetic damage directly or indirectly by various mechanisms. However, no single assay is capable of detecting all relevant genotoxic agents. Therefore, the usual approach should be to carry out in the battery of *in vitro* and *in vivo* assays for genotoxicity. Generally, the following standard battery for genotoxicity testing for pharmaceuticals is recommended (ICH Harmonized Tripartite Guideline, 1997): 1) Assay for gene mutation in bacteria; 2) An *in vitro* assay of cytogenetic evaluation on chromosomal damage with mammalian cells; 3) *In vivo* assay for chromosomal damage using rodent hematopoietic cells. Therefore, to investigate the genotoxic profiles of RVS, we carried out the Ames assay, *in vitro* chromosomal aberration test using Chinese hamster lung (CHL) cells, and *in vivo* micronucleus assay using male ICR mice.

## MATERIALS AND METHODS

***Test item material and chemicals.*** Water and ethanol extracts from *Rhus verniciflua* Stokes (RVS) were obtained in powder from Korea Food Research Institute (Sungnam, Korea). Freeze-dried water and ethanol extracts were dissolved in distilled water or DMSO, respectively, to make 10-fold (for Ames assay) or 100-fold (for *in vitro* chromosomal aberration assay) stock solution immediately before use and serially diluted to the appropriate concentrations. The test items were freely soluble up to the highest stock concentration. Most chemicals including positive controls such as 4-nitroquinoline 1-oxide (NQO) and cyclophosphamide were obtained from Sigma (St. Louis, MO). MEM medium, fetal bovine serum, and penicillin-streptomycin were purchased from GIBCO-Invitrogen (Carlsbad, CA). S9, which was prepared from male Sprague-Dawley rats induced with Aroclor 1254, was from Molecular Toxicology Inc. (Boone, NC) and cofactor for S9 mix was from Wako Pure Chem. Ind., Ltd. (Japan).

***Animals.*** Approximately 5.5-week old specific pathogen free male ICR mice weighing 25.4~29.3 g were obtained from Orient Co., Ltd. (Seoul, Korea). Animals were housed in polycarbonate cages. An ambient temperature of $25 \pm 2^{o}C$, relative humidity of $50 \pm 2\%$, and photoperiod of 12 h was maintained throughout the study. Commercial pellet diet (PMI Nutrition International, Richmond, IN) and water were provided *ad libitum*. Clinical signs of animals were checked and recorded once a day for 11 days of quarantine and acclimatization. All animals used in this study were cared in accordance with the principles outlined in the "Guide for the Care and Use of Laboratory Animals", a NIH publication (earned AAALAC International accreditation in 1998).

***Cell culture.*** *In vitro* chromosomal aberration assay was performed using Chinese Hamster Lung cells (CHL) (Hong *et al.*, 2005), which were obtained from American Type Culture Collection (Manassas, VA). The cells were cultured in Minimum Essential Medium supplemented with 100 U penicillin, 100 mg/ml streptomycin, 2 mM L-glutamine, and 10% fetal bovine serum. Sub-culture was conducted every 2~3 days so as to prevent overgrowth.

***Ames assay.*** *Salmonella typhimurium* strains TA 98 and TA1537 (detect frame-shift mutagens), and strains TA100, TA1535 and *Escherichia coli* WP2 uvrA (detect base-pair substitution mutagens) were used as tester strains. All of the tester strains were purchased from Molecular Toxicology Inc. (Boone, NC). The mutation assay was performed according to the method of Maron and Ames (Maron and Ames, 1983). A 0.1 ml aliquot of *Rhus verniciflua* Stokes (RVS) containing 313~5000 mg per plate, 0.5 ml of S9 mix (or sodium-phosphate buffer, pH 7.4 for S9 negative group), and 0.1 ml inoculum of the tester strain were added to each tube containing 2 ml of top agar. The contents of test tubes were mixed well and the mixtures were poured onto the Vogel-Bonner minimal agar plates. Plates were incubated at $37^{o}C$ for 48 h. Triplicate plates were run for each assay.

***In vitro chromosomal aberration assay.*** *In vitro* chromosomal aberration assay was performed using Chinese hamster lung fibroblast cells (CHL) as described by Ishidate *et al*. (1981) and Dean and Danford (1984) with minor modifications. The CHL cells have a stable kayrotype, a short generation time and are easy to maintain. The assay was consisted of short-term (6 h) and continuous (24 h) treatments. Approximately 22 hours after the start of the treatment, colchicine was added to each culture at a final concentration of 1 $\mu$M. The slides of CHL cells were prepared following the hypotonic-methanol-glacial acetic acid-flame drying-Giemsa schedule for metaphase plate analysis. The 200 metaphases (100 metaphases from each duplicate culture) were selected and analyzed for each treatment group under 1000 × magnification using a light microscope (Nikon Microphoto). The results were expressed as mean aberrant metaphases excluding gaps per 100 metaphases. Regardless of the presence of aberration, additional 100 metaphases were examined to determine the frequency of polyploid (PP) and endoreduplication (ER).

***In vivo micronucleus assay.*** RVS was administered by gavage to groups of six mice at doses of 500, 1000 and 2000 mg/kg. Mice in negative control group received only the vehicle (distilled water for water extracts; 20% propyleneglycol for ethanol extracts) by gavage. Cyclophosphamide in normal saline (10 ml/kg) was administered to six mice by intraperitoneal injection at 70 mg/kg and served as positive control. Mice were euthanized 24 h after last administration, which showed the peak induction of micronuclei. Bone marrow was prepared as previously described (Kim *et al*., 2002) for evaluation. Following the sacrifice of animals, one femur was excised intact. The bone marrow was expelled from the cavity by repeated gentle aspirations and flushings with fetal bovine serum. The cell suspension was centrifuged at approximately 1000 rpm for 5 min. The supernatant was decanted and the pellet was resuspended in a small volume of serum. At least two slides of cell suspension per animal were made. The air-dried slides were stained with May-Grunwald and Giemsa. Smears were allowed to dry overnight before being coverslipped with mounting medium. Slides were then examined under 1000 × magnification. Small round or oval shaped bodies, size of about 1/5 to 1/20 of the diameter of polychromatic erythrocyte (PCE), were counted as micronuclei. A total of 2000 PCEs were scored per animal by the same observer for determining the frequencies of micronucleated polychromatic erythrocytes (MNPCEs). PCE/(PCE+NCE) ratio was calculated by counting 500 cells.

***Statistical analysis.*** The statistical analyses for *in vitro* chromosomal aberration results were conducted using Statistical Analysis System (SAS) program according to Richardson *et al*. (1989). The number of aberrant metaphases (excluding gaps) and number of [PP+ER] were analyzed. The $\chi^2$-test and Fisher's exact test were performed for comparison of the vehicle control and test item-treated groups. The comparison of the vehicle and positive control groups was performed using Fisher's exact test. Differences were regarded as statistically significant, if $P < 0.05$. Statistical evaluation of the *in vivo* micronucleus results was performed according to Lovell *et al*. (1989) with minor modification. Data showing heterogeneous variances were analyzed using Kruskal Wallis analysis of variance followed by multiple comparisons using the Dunnett's test. The study was accepted when all of the PCE/(PCE+NCE) ratio were greater than 0.1 (Heddle *et al*., 1984). The result was judged as positive when there was a statistically significant and dose-related increase or a reproducible increase in the frequency of MNPCEs (*in vivo* MN assay) or aberrant metaphases (*in vitro* CA assay) at least at one dose level. The result of the statistical evaluation was regarded significant when the *P* value was less than 0.05. No statistical analysis was performed on Ames results.

## RESULTS

***Ames assay of Rhus verniciflua Stokes (RVS).*** Histidine-requiring mutants of *S. typhimurium* (TA98, TA100, TA1535 and TA1537) and tryptophan-requiring mutants of *E. coli* WP2 *uvrA* with and without metabolic activation (S9) were used for point-mutation tests. The dose range-finding test was performed to determine the highest concentration for Ames test. No toxicity was observed after the addition of water and ethanol extracts of RVS up to 5000 mg/plate in any strains. Thus, the mutagenicity of RVS was evaluated up to a maximum dose of 5000 mg/plate. The results of Ames test of RVS are shown in Table 1. There was no increase in the number of revertant colonies compared to its negative control at any dose in TA100, TA1535 and WP2 *uvrA* strains. However, in TA98 and TA1537 strains, the addition of RVS water extracts at doses from 313 up to 5000 mg/plate induced an increase more than 2-fold over vehicle control in the number of revertant colonies with a maximum of 4.9-fold increase in the absence of S9 mix. RVS ethanol extracts also caused the maximum 5.8- and 4.5-fold increases in the number of revertant colonies at 5000 mg/plate in the presence of S9 mix in TA98 and TA1537, respectively. Reproducibility was confirmed with the data from the dose range-finding study and main study. The number of revertant colonies in both the negative and positive controls was within the range based on our historical data (data not shown).

***In vitro chromosomal aberration assay of Rhus verniciflua Stokes (RVS).*** From the two dose range-finding tests with the highest concentration of 5 mg/ml, dose range of *in vitro* CA test was designed to contain the concentration showing about 50% of the relative cell count (RCC), which was determined by comparing cell counts in test item and vehicle control cultures. Duplicate treatments were conducted for each concentration. In the case of continuous treatment without S9, both water and ethanol extracts of RVS induced the increase in structural chromosomal aberrations at the highest concentration (Table 2). Also in the short-term treatment, structural aberrations were induced by both water and ethanol extracts of RVS regardless of S9 mix application (Table 2 and Table 3). In particular, a dose-related increase was observed when water extract of RVS was treated for 6-h treatment without S9 mix. The number of metaphases with structural aberrations in the vehicle and positive control groups were within the range established in historical data. Thus, RVS was considered to be clastogenic in this assay at up to the highest feasible concentration that could be evaluated. On the other hand, no statistically significant increase in the number of metaphases with numerical aberrations was observed at any concentration tested (data not shown).

**Table 1.** Results observed of water and ethanol extracts from *Rhus verniciflua* Stokes (RVS) in Ames test

| Test strains | Dose (μg/plate) | Water extract | | 70% ethanol extract | |
|---|---|---|---|---|---|
| | | Colonies/plate (Mean) [Factor][a] | | Colonies/plate (Mean) [Factor] | |
| | | Without S-9 mix | With S-9 mix | Without S-9 mix | With S-9 mix |
| TA100 | 0 | 127 ± 7 | 124 ± 9 | 121 ± 9 | 122 ± 6 |
| | 313 | 135 ± 11 [1.1] | 138 ± 6 [1.1] | 114 ± 10 [0.9] | 129 ± 10 [1.1] |
| | 625 | 137 ± 15 [1.1] | 172 ± 3 [1.4] | 142 ± 15 [1.2] | 145 ± 6 [1.2] |
| | 1250 | 155 ± 11 [1.2] | 188 ± 18 [1.5] | 132 ± 20 [1.1] | 161 ± 1 [1.3] |
| | 2500 | 184 ± 12 [1.4] | 192 ± 3 [1.5] | 144 ± 2 [1.2] | 189 ± 4 [1.5] |
| | 5000 | 211 ± 20 [1.7] | 223 ± 16 [1.8] | 132 ± 17 [1.1] | 168 ± 19 [1.4] |
| TA1535 | 0 | 15 ± 4 | 14 ± 3 | 14 ± 3 | 11 ± 1 |
| | 313 | 12 ± 2 [0.8] | 12 ± 2 [0.9] | 14 ± 4 [1.0] | 12 ± 2 [1.1] |
| | 625 | 15 ± 3 [1.0] | 13 ± 3 [0.9] | 14 ± 1 [1.0] | 11 ± 1 [1.0] |
| | 1250 | 15 ± 1 [1.0] | 13 ± 3 [0.9] | 13 ± 2 [0.9] | 11 ± 1 [1.0] |
| | 2500 | 14 ± 2 [0.9] | 12 ± 3 [0.9] | 11 ± 2 [0.8] | 12 ± 2 [1.1] |
| | 5000 | 14 ± 1 [0.9] | 11 ± 2 [0.8] | 8 ± 1 [0.6] | 11 ± 2 [1.0] |
| TA98 | 0 | 27 ± 3 | 33 ± 4 | 18 ± 3 | 25 ± 3 |
| | 313 | 33 ± 2 [1.2] | 43 ± 2 [1.3] | 26 ± 3 [1.4] | 55 ± 4 [2.2] |
| | 625 | 32 ± 2 [1.2] | 50 ± 4 [1.5] | 35 ± 4 [1.9] | 56 ± 3 [2.2] |
| | 1250 | 35 ± 4 [1.3] | 64 ± 3 [1.9] | 56 ± 6 [3.1] | 85 ± 13 [3.4] |
| | 2500 | 47 ± 1 [1.7] | 72 ± 6 [2.2] | 75 ± 5 [4.2] | 118 ± 11 [4.7] |
| | 5000 | 56 ± 4 [2.1] | 79 ± 1 [2.4] | 85 ± 4 [4.7] | 144 ± 12 [5.8] |
| TA1537 | 0 | 9 ± 1 | 16 ± 3 | 9 ± 2 | 14 ± 2 |
| | 313 | 15 ± 2 [1.7] | 19 ± 2 [1.2] | 23 ± 2 [2.6] | 27 ± 3 [1.9] |
| | 625 | 21 ± 1 [2.3] | 23 ± 3 [1.4] | 25 ± 3 [2.8] | 31 ± 2 [2.2] |
| | 1250 | 29 ± 6 [3.2] | 40 ± 3 [2.5] | 33 ± 1 [3.7] | 46 ± 3 [3.3] |
| | 2500 | 37 ± 3 [4.1] | 39 ± 3 [2.4] | 30 ± 2 [3.3] | 50 ± 3 [3.6] |
| | 5000 | 44 ± 5 [4.9] | 49 ± 4 [3.1] | 27 ± 8 [3.0] | 63 ± 4 [4.5] |
| *E. coli* WP2 uvrA | 0 | 20 ± 3 | 23 ± 2 | 17 ± 3 | 19 ± 1 |
| | 313 | 23 ± 3 [1.2] | 24 ± 2 [1.0] | 14 ± 2 [0.8] | 21 ± 1 [1.1] |
| | 625 | 23 ± 3 [1.2] | 32 ± 3 [1.4] | 19 ± 2 [1.1] | 22 ± 2 [1.2] |
| | 1250 | 24 ± 3 [1.2] | 24 ± 3 [1.0] | 23 ± 1 [1.4] | 29 ± 3 [1.5] |
| | 2500 | 26 ± 3 [1.3] | 26 ± 5 [1.1] | 27 ± 2 [1.6] | 29 ± 3 [1.5] |
| | 5000 | 28 ± 2 [1.4] | 30 ± 1 [1.3] | 21 ± 2 [1.2] | 26 ± 2 [1.4] |
| Positive controls | | | | | |
| TA100 | SA | 411 ± 32 [3.2] | | 464 ± 8 [3.8] | |
| TA1535 | SA | 375 ± 19 [25.0] | | 402 ± 7 [28.7] | |
| TA98 | 4NQO | 226 ± 13 [8.4] | | 192 ± 7 [10.7] | |
| TA1537 | 9-AA | 135 ± 22 [15.0] | | 152 ± 35 [16.9] | |
| WP2 | 4NQO | 277 ± 16 [13.9] | | 121 ± 17 [7.1] | |
| TA100 | 2-AA | | 329 ± 34 [2.7] | | 415 ± 17 [3.4] |
| TA1535 | 2-AA | | 215 ± 27 [15.4] | | 371 ± 8 [33.7] |
| TA98 | 2-AA | 32 ± 2 [1.2] | 427 ± 29 [12.9] | 23 ± 3 [1.3] | 403 ± 14 [16.1] |
| TA1537 | 2-AA | | 225 ± 22 [14.1] | | 403 ± 14 [28.8] |
| WP2 | 2-AA | | 234 ± 33 [10.2] | | 292 ± 7 [15.4] |

[a]No. of colonies of treated plate/No. of colonies of negative control plate; Data were expressed as the mean numbers of colonies ± S.D. from triplicate plates/concentration.
SA, Sodium azide (0.5 mg/plate); 9-AA, 9-Aminoacridine (50 mg/plate); 4NQO, 4-Nitroquinoline-1-oxide (0.5 mg/plate); 2-AA, 2-Aminoanthracene (0.4 mg/plate for TA100 and TA98; 2 mg/plate for TA1535 and TA1537; 4 mg/plate for WP2).

***In vivo* micronucleus assay of *Rhus verniciflua* Stokes (RVS).** For *in vivo* micronucleus test, the dose levels were determined by a preliminary dose-range finding test, in which, using the algorithm of Path/Tox system (version 4.2.2), four males and four females were assigned to each group on the day before administration. Animals were dosed with the test item at dose levels of 250, 500, 1000 and 2000 mg/kg for two consecutive days. No mortality occurred even at 2000 mg/kg in both male and female mice. Since there was no significant toxicological difference between both sexes, male mice, which were found to have the higher sus-

**Table 2.** *In vitro* chromosome aberration test results without metabolic activation

| Dose (μg/ml) | Treatment time (h)[a] | Mean[b] aberrant metaphase | Mean total aberrations | Number of findings/100 metaphase | | | | | | RCC (%)[c] |
|---|---|---|---|---|---|---|---|---|---|---|
| | | | | Gap | cht type | | ctd type | | Other | |
| | | | | | brk | exc | brk | exc | | |
| Water extract from from *Rhus verniciflua* Stokes (RVS) | | | | | | | | | | |
| 0 | 6-18 | 0.0 | 0.0 | 0 | 0 | 0 | 0 | 0 | 0 | 100 |
| 125 | 6-18 | 0.0 | 0.0 | 0.5 | 0 | 0 | 0 | 0 | 0 | 101 |
| 250 | 6-18 | 8.5**[d] | 11.0 | 2 | 0.5 | 1 | 1.5 | 5.5 | 2.5 | 66 |
| 500 | 6-18 | 18.5** | 30.0 | 2 | 0.5 | 1 | 9 | 16.5 | 3.0 | 35 |
| 0 | 24-0 | 0.5 | 0.5 | 0 | 0 | 0.5 | 0 | 0 | 0 | 100 |
| 107.5 | 24-0 | 0.0 | 0.0 | 1 | 0 | 0 | 0 | 0 | 0 | 60 |
| 215 | 24-0 | 1.0 | 2.0 | 1 | 0 | 0 | 0 | 2 | 0 | 43 |
| 430 | 24-0 | 19.0** | 29.5 | 5 | 0.5 | 0 | 6.5 | 20 | 2.5 | 40 |
| Ethanol extract from from *Rhus verniciflua* Stokes (RVS) | | | | | | | | | | |
| 0 | 6-18 | 0.0 | 0.0 | 0.5 | 0 | 0 | 0 | 0 | 0 | 100 |
| 37.5 | 6-18 | 0.0 | 0.0 | 1.5 | 0 | 0 | 0 | 0 | 0 | 92 |
| 75 | 6-18 | 1.0 | 1.0 | 0 | 0 | 0 | 1 | 0 | 0 | 92 |
| 150 | 6-18 | 5.5** | 6.0 | 1.5 | 0 | 1 | 1 | 1.5 | 2.5 | 53 |
| 0 | 24-0 | 0.0 | 0.0 | 0 | 0 | 0 | 0 | 0 | 0 | 100 |
| 20 | 24-0 | 0.0 | 0.0 | 0 | 0 | 0 | 0 | 0 | 0 | 84 |
| 40 | 24-0 | 0.5 | 1.0 | 2 | 0 | 0 | 1 | 0 | 0 | 78 |
| 80 | 24-0 | 9.5** | 15.0 | 10.5 | 1.5 | 0 | 13 | 0 | 1.5 | 41 |
| Positive control (EMS)[f] | | | | | | | | | | |
| 800 | 6-18 | 19.0**[e] | 23.5 | 2 | 0 | 0 | 4.5 | 18 | 1 | 67 |
| 600 | 24-0 | 22.5** | 33.0 | 2.5 | 0 | 0.5 | 6 | 25 | 1.5 | 38 |

[a]Time, Chemical treatment time-recovery time.
[b]Gaps excluded; the mean aberrant metaphases on two slides from two different cultures; 100 metaphases were examined per culture.
[c]RCC = (Cell counts of treated flask/Cell counts of untreated flask)×100.
[d]$\chi^2$-test and Fisher's exact test; $^{**}P < 0.01$.
[e]Fisher's exact test; $^{**}P < 0.01$.
[f]EMS, Ethylmethanesulfonate, dissolved in distilled water.
Abbreviations: RCC, relative cell count; chr, chromosome; ctd, chromatid; brk, break; exc, exchange.

ceptibility to clastogens (The Collaborative Study Group for the Micronucleus Test, 1986), were used for *in vivo* micronucleus test. Water and ethanol extracts of RVS were orally administered two times at 24 hr intervals at doses of 0, 500, 1000 and 2000 mg/kg using 7-week old male mice. Table 4 shows the incidence of micronucleated polychromatic erythrocytes (MNPCEs) per 2000 polychromatic erythrocytes (PCEs). There was a weak but statistically significant increase in number of MNPCEs in mice treated with water extract at 2000 mg/kg. Although slight decreases were observed in mean numbers of PCEs per 500 erythrocytes (PCE/[PCE+NCE]), an indicator of cytotoxicity, in groups treated with water extract, none of these changes was statistically significant. Conversely, ethanol extract of RVS being given orally at 2 daily doses up to 2000 mg/kg did not induce any statistically significant changes in the incidence of MNPCEs when compared to vehicle control. In addition, no remarkable effects of ethanol extract were seen on PCE/[PCE+NCE] ratio. The incidence of MNPCEs in both the negative and positive controls were within the range based on our historical data.

## DISCUSSION

*Rhus verniciflua* Stokes (RVS) were previously found to have anti-AIDS, a strong antioxidant and immune-enhancing activities (Miller *et al.*, 1996), although it contains toxic substances that can cause allergic irritation to some people. Despite its increasing use, very little data are available in literature on the potential genotoxicity of RVS. Thus, the genotoxic effects of RVS were evaluated with standard 3-test battery recommended by the International Conferences on Harmonization (ICH) of Technical Requirements for Registration of Pharmaceuticals for Human Use: (1) Bacterial reverse mutation test (Ames test), (2) *In vitro* chromosome aberration test using CHL cells and (3) *In vivo* micronucleus test using ICR mice.

In *Salmonella typhimurium* strains using Ames test, RVS was shown to be a strong mutagenic agent. Especially, it caused a dramatic increase in the reversion fre-

**Table 3.** *In vitro* chromosome aberration test results with metabolic activation

| Dose (μg/ml) | Treatment time (h)[a] | Mean[b] aberrant metaphase | Mean total aberrations | Number of findings/100 metaphase | | | | | | RCC (%)[c] |
|---|---|---|---|---|---|---|---|---|---|---|
| | | | | Gap | cht type | | ctd type | | Other | |
| | | | | | brk | exc | brk | exc | | |
| Water extract from *Rhus verniciflua* Stokes (RVS) | | | | | | | | | | |
| 0 | 6-18 | 0.0 | 0.0 | 0.5 | 0 | 0 | 0 | 0 | 0 | 100 |
| 187.5 | 6-18 | 0.0 | 0.0 | 0.5 | 0 | 0 | 0 | 0 | 0 | 79 |
| 375 | 6-18 | 0.0 | 0.0 | 0.5 | 0 | 0 | 0 | 0 | 0 | 118 |
| 750 | 6-18 | 4.0**[d] | 6.5 | 1 | 0 | 0 | 0.5 | 4 | 2 | 53 |
| Ethanol extract from *Rhus verniciflua* Stokes (RVS) | | | | | | | | | | |
| 0 | 6-18 | 0.0 | 0.0 | 0 | 0 | 0 | 0 | 0 | 0 | 100 |
| 42.5 | 6-18 | 0.0 | 0.0 | 2 | 0 | 0 | 0 | 0 | 0 | 96 |
| 85 | 6-18 | 1.5 | 1.5 | 2.5 | 0 | 0 | 1.5 | 0 | 0 | 89 |
| 170 | 6-18 | 5.5** | 9.0 | 4 | 0.5 | 0 | 3 | 4.5 | 1 | 90 |
| Positive control (CPA) | | | | | | | | | | |
| 6 | 24-0 | 12.5**[e] | 17.5 | 1 | 0 | 0 | 3 | 14.5 | 0 | 67 |

[a]Time, Chemical treatment time-recovery time.
[b]Gaps excluded; the mean aberrant metaphases on two slides from two different cultures; 100 metaphases were examined per culture.
[c]RCC = (Cell counts of treated flask/Cell counts of untreated flask) × 100.
[d]$\chi^2$-test and Fisher's exact test; *$P < 0.05$, **$P < 0.01$.
[e]Fisher's exact test; **$P < 0.01$.
[f]CPA, cyclophosphamide monohydrate, dissolved in distilled water.
Abbreviation: RCC, Relative cell count; chr, chromosome; ctd, chromatid; brk, break; exc, exchange.

**Table 4.** Effect of water and ethanol extracts from *Rhus verniciflua* Stokes (RVS) on *in vivo* micronucleus assay in ICR male mice

| Dose (μg/kg) | No. of animal | MNPCE/2000 PCEs (Mean ± S.D.) | PCE/(PCE + NCE) (Mean ± S.D.) |
|---|---|---|---|
| Water extract from *Rhus verniciflua* Stokes (RVS) | | | |
| Water | 6 | 1.50 ± 0.55 | 0.47 ± 0.07 |
| 500 | 6 | 1.33 ± 0.52 | 0.41 ± 0.11 |
| 1000 | 6 | 2.00 ± 1.41 | 0.43 ± 0.07 |
| 2000 | 6 | 3.33 ± 1.03*[a] | 0.39 ± 0.06 |
| Positive control (CPA, cyclophosphamide monohydrate) | | | |
| 70 | 6 | 69.00 ± 4.60**[b] | 0.36 ± 0.07*[c] |
| 70% ethanol extract from *Rhus verniciflua* Stokes (RVS) | | | |
| Untreated | 6 | 3.67 ± 0.82 | 0.49 ± 0.05 |
| Proplyene glycol | 6 | 4.17 ± 1.47 | 0.43 ± 0.07 |
| 500 | 6 | 2.67 ± 0.82 | 0.41 ± 0.04 |
| 1000 | 6 | 3.50 ± 1.76 | 0.44 ± 0.08 |
| 2000 | 6 | 4.67 ± 1.37 | 0.46 ± 0.03 |
| Positive control (CPA, cyclophosphamide monohydrate) | | | |
| 70 | 6 | 84.83 ± 10.96**[b] | 0.44 ± 0.04*[c] |

**Significantly different from the vehicle control at $P < 0.01$.
*Significantly different from the vehicle control at $P < 0.05$.
[a]Kruskal-Wallis' H-test/Dunnett's test
[b]Mann-Whitney's U-test.
[c]Student's t-test.
Abbreviations: PCE, Polychromatic erythrocyte; NCE, Normochromatic erythrocyte; MNPCE, PCE with one or more micronuclei.

quency of TA98 and TA1537 strains. Since the tester strains TA98 and TA1537 detect frame-shift mutagens, these results imply that RVS may induce the bacterial reversion by frame-shift mutation. The application of metabolic activation system (S9 mix) had little effect on the mutagenic potential of RVS. Contrary to our results, other laboratory found that the addition of 1.0 mg/plate of the methanolic extract or the ethylacetate fraction of the *Rhus verniciflua* heartwood extract potentially inhibited the mutagenicity by aflatoxin $B_1$ (Park *et al*., 2004). However, they performed Ames test only using TA100, in which we also found no mutagenic potential of RVS.

Moreover, the mutagenicity of RVS itself was not investigated in their study.

*In vitro* chromosome aberration test using CHL cells was performed to investigate if RVS affects the consistent genotoxicity tendency. Both water and ethanol extracts induced significant increases in the number of metaphases with structural aberrations under present experimental conditions. Water extracts appeared to induce more structural aberration than ethanol extract. The cytotoxicity was induced in CHL cells at much lower concentration of ethanol extract compared to water extract. Thus, the concentration of active ingredient to induce structural aberration in ethanol extract is likely to be lower than that of water extract. On the other hand, the analysis of standard genotoxicity assay data that has been conducted for safety evaluation of chemical compounds usually reveals a fairly high percentage of positive results associated with cytotoxicity in the *in vitro* chromosomal aberration assay (Galloway, 2000; Muller and Sofuni, 2000). In general, 50% cell growth inhibition concentration is used as a maximum concentration for *in vitro* chromosomal aberration assay. Muller *et al*. (2000) suggested that lowering the upper limits of test compound concentration irrespective of cytotoxicity may prove useful to ensure a sufficient reliability of genotoxicity test with mammalian cells *in vitro*. In this study, both water and ethanol extracts from RVS were found as positive in the chromosomal aberration assay mostly at concentrations showing the cell survival less than 60%. Thus, we cannot exclude the possibility that RVS is the cytotoxic clastogens, resulting in false-positive response.

Further, to confirm the *in vitro* genotoxicity results of RVS, *in vivo* micronucleus assay was additionally performed using male ICR mice. Although the current preferred method of staining for MN assay is acridine orange staining (Tinwell and Ashby, 1989), Giemsa staining was chosen because it is a permanent and does not fade even when exposed to strong light during the scoring period. Giemsa stain is acceptable by an international expert group that recently reviewed *in vivo* rodent erythrocyte micronucleus assay in the International Workshop on Genotoxicity Test Procedures (Hayashi *et al*., 2000). Based on the information of kinetics of erythropoiesis and the recommendations and observations made by several laboratories (Henderson *et al*., 1984; Krishna and Hayashi, 2000; Salamone and Heddle, 1983), mice were euthanized 24 h after last administration with extracts from RVS. The results of this assay demonstrated that, under the conditions employed, ethanol extracts of RVS at doses of up to 2000 mg/kg did not induce any statistically significant changes in the incidence of micronucleated bone marrow cells, suggesting that it is not clastogenic after *in vivo* exposure. Conversely, water extracts from RVS induced a weak but statistically significant micronuclei increase on mouse bone marrow cells.

Although we did not determine which active ingredients were putatively responsible for the genotoxic property in the extracts, biological activities of urushiol and flavonoids from RVS have been previously reported (Jung, 1998). Urushiols, which are the skin-irritating poison found in plants, have been reported to have an anticancer activity to human cancer cells (Hong *et al*., 1999). Also, flavonoids purified from RVS actively inhibited cell growth and induce apoptosis in mouse tumorigenic hepatic cells (Son *et al*., 2005) and human osteosarcoma cells (Jang *et al*., 2005). Lee *et al*. (2002) isolated fustin, fisetin, sulfuretin, and butein in the flavonoid fractions prepared from a crude acetone extract of RVS. Most of these substances are believed to exert their chemotherapeutic activity by blocking the cell cycle progression and triggering apoptotic cell death. Butein was found to inhibit the activity of tyrosine kinases (Yang *et al*., 1998), which are known to be overexpressed in many cancer tissues. Fisetin mediated antitumor and anti-inflammatory effects through modulation of NF-κB pathways (Sung *et al*., 2007). Sulfuretin prevented rheumatoid syndromes by inhibiting reactive oxygen species (Choi *et al*., 2003).

Contrary to these reported anticarcinogenic activity, it has been also known that many flavonoids could be carcinogenic or pro-oxidants to DNA at certain concentrations (Johnson and Loo, 2000; MacGregor and Jurd, 1978). In fact, some of flavonoids such as procyanidin B4, catechin, and gallic acid could prevent oxidative damage to cellular DNA at low concentration, while, at higher concentration, these compounds might induce cellular DNA damage, (Fan and Lou, 2004). Park *et al*. (2004) suggested that, among of the flavonoids from RVS, fustin might have both mutagenicity and antimutagenicity. Our results lead to conclusion that RVS acts as a genotoxic material based on the available *in vitro* and *in vivo* results. This conclusion raises the necessity of identification of a genotoxic mechanism for RVS prior to its development as a cancer chemopreventive agent and therapeutic substance. Thus, further detailed experiments will be needed to determine the active ingredient responsible for inducing genotoxic effect and to unravel exactly its genotoxic mechanism.
